# Directed Evolution of AtMP2 Peptide: Unlocking Enhanced Antibacterial Potential from *Anabas testudineus*

**DOI:** 10.3390/molecules30234590

**Published:** 2025-11-28

**Authors:** Li Ting Lee, Arnold Ang, Ahmed Najm, Adura Mohd Adnan, Akram Mohd Nordin, Ibrahim Mahmood, Sarantuya Dunkhorol, Shazrul Fazry, Douglas Law

**Affiliations:** 1 Faculty of Health and Life Sciences, Inti International University, Nilai 71800, Negeri Sembilan, Malaysia; 2 Medical Laboratory Science, Lebanese French University, Kurdistan Region, Erbil 44001, Iraq; 3 Department of Food Science, Faculty of Science and Technology, Universiti Kebangsaan Malaysia, Bangi 43600, Selangor, Malaysia; 4 Department of Biological Sciences and Biotechnology, Faculty of Science and Technology, Universiti Kebangsaan Malaysia, Bangi 43600, Selangor, Malaysia; 5 Dentistry Department, Al-Rafidain University College, Baghdad 46036, Iraq; 6 School of Technology in Darkhan, Mongolia University of Science and Technology, Darkhan 61110, Darkhan-Uul Aimag, Mongolia; 7 Faculty of Health Sciences, Shinawatra University, Pathum Thani 12160, Thailand; 8 Faculty of Science, Assam Downtown University, Guwahati 781026, Assam, India

**Keywords:** antimicrobial peptides, fish proteins, *Anabas testudineus*, drug resistance, human health

## Abstract

Previous studies have shown that AtMP1 and AtMP2, antimicrobial peptides found in the climbing perch (*Anabas testudineus*), possess antibacterial and anticancer properties. This study aimed to determine whether modified forms of the AtMP2 peptide could enhance its antimicrobial activity. In the research study, the epidermal mucus of *Anabas testudineus* was extracted and tested to contain antibacterial and anticancer properties. Two peptides were initially extracted from the freshwater fish *Anabas testudineus*; however, the focus was placed on AtMP2 to evaluate its potential for enhanced antibacterial activity. Systematic-directed evolution was used to generate AtMP2 varieties. The AtMP2 varieties were characterized using APD3, CAMP, and AMPFun. Based on the characterization, two peptides, AtMP2-1 and AtMP2-2, were selected for synthesis. AtMP2-1 and AtMP2-2 demonstrated higher antimicrobial activity against Gram-positive than Gram-negative bacteria compared to the AtMP2 peptide, based on Minimum Inhibitory Concentration (MIC) determination and Kirby–Bauer Disk Diffusion analysis. For the SRB cytotoxicity analysis using the HS-27 and Vero cell lines, both AtMP2-1 and AtMP2-2 are safe for human use at 20 μg/mL. ZDOCK and HPEPDOCK servers were used to evaluate the binding affinity between AtMP2-1 and AtMP2-2 peptides and proteins involved in the bacterial cell death cycle. The results showed varying docking scores, with more negative values indicating stronger binding interactions, particularly with proteins such as SecA, RpoB, GyrA, ClpP, and MetG. The study concluded that modified peptides derived from *Anabas testudineus* show enhanced antibacterial properties and minimal cytotoxicity, making them potential alternatives to traditional antibiotics. Future research should investigate the specific biochemical pathways affected by these AMPs to understand their mechanisms of action better and explore their potential as therapeutic agents, particularly in the treatment of bacterial infections, wound healing, and cancer therapy.

## 1. Introduction

Antibiotic use is widespread and often misused. Although antibiotics have transformed medicine and saved millions of lives, their effectiveness is declining due to the global rise in antibiotic-resistant bacteria. Despite the success of antibiotics in treating infections for decades, bacterial infections are once again becoming a global health threat due to increasing resistance [1]. The fact that there are many more bacteria in the colon than cells in the body demonstrates how antibiotics affect the entire body. Human health depends on the gut microbiome [2]. Antibiotics frequently kill good flora and cause opportunistic infections in areas such as the vagina and gut [2]. Antibiotic prescriptions for diseases are believed to be unnecessary in 50% of cases [3]. Studies estimate that up to 50% of antibiotic prescriptions are inappropriate, with errors in agent selection, treatment duration, or indication [4,5].

Small peptides known as antimicrobial peptides (AMPs) are produced by various organisms, including animals, plants, fungi, and bacteria. These molecules are vital to the innate immune system and play a key role in defending against microbial infections [4]. They function by compromising the integrity of microbial cell membranes, resulting in cell death. Due to this mechanism, AMPs have garnered increased interest as potential substitutes for traditional antibiotics, owing to their broad-spectrum activity and low risk of microbial resistance [6]. Many AMPs have positive charges that enable them to bind to the negatively charged elements of microbial membranes, such as the lipopolysaccharides found in bacteria. This binding may result in membrane pores, allowing vital intracellular components to seep out and ultimately cause cell death [7]. Certain AMPs are currently being studied to create novel AMPs with enhanced efficacy and safety profiles [8].

*Anabas testudineus*, commonly known as the climbing perch, is a freshwater fish native to Southeast Asia. AMPs have been found in several *A. testudineus* tissues and bodily fluids, including skin mucus [9]. Studies conducted by Najm (2021) confirmed that the fish *Anabas testudineus* produces AMPs, as the mucus collected from the fish was fractionated and tested, demonstrating antibacterial activity [10]. Two peptides, AtMP1 and AtMP2, were synthetically produced and initially highlighted for their antimicrobial properties [10]. Further testing revealed that these peptides possess significant anticancer properties, promising potential for the development of highly effective and specifically targeted cancer therapies based on antimicrobial peptides.

Therefore, this study aimed to evaluate whether modified forms of the AtMP2 peptide, derived from the epidermal mucus of Anabas testudineus, exhibit enhanced antimicrobial activity against both Gram-positive and Gram-negative bacteria [11]. The peptides were designed through systematic directed evolution via amino acid substitution and selected based on predicted bioactivity scores. The study also assessed their cytotoxicity in human cell lines and performed molecular docking to identify potential interactions with bacterial target proteins involved in cell death [12]. These findings contribute to the development of safer and more effective peptide-based antimicrobial agents.

## 2. Results and Discussion

### 2.1. Selection of Peptides

The top 20 peptides were chosen based on their antimicrobial activity, hydrophobicity, total net charge, and anticancer properties (Table 1). The antimicrobial activity of Gram-positive and Gram-negative bacteria was investigated using AMPFun after the systematic single amino acid replacement.

AMP predictions were obtained from multiple databases (APD3, CAMPR3, AMPFun) to account for variability across classifiers. While CAMPR3 yielded scores below the 0.5 AMP threshold, AMPFun produced high probability values (~0.96–0.98). These differences reflect distinct algorithmic approaches and training datasets. Importantly, our experimental data (disk diffusion and MIC assays) provide evidence of antibacterial activity. However, further validation, such as time-kill and membrane interaction assays, is required to confirm potency and mechanism.

The selection and evaluation of antimicrobial peptides (AMPs) are critical in identifying potential therapeutic candidates to combat bacterial infections. Table 1 focused on the top 20 peptides assessed for their antimicrobial properties, hydrophobicity, total net charge, and anticancer properties. The AMPFun database was utilized to predict the antimicrobial activity of peptides against Gram-positive and Gram-negative bacteria, offering a detailed analysis of each peptide’s potential.

Table 1 presents predicted quantitative data on the antimicrobial potential of the tested peptides. The column ‘Antimicrobial Activity’ displays the predicted antimicrobial activity scores for each peptide, with higher values indicating more substantial antimicrobial potential. The “Gram+” and “Gram−” columns represent the expected effectiveness of each peptide against Gram-positive and Gram-negative bacteria, respectively, with values ranging from 0 to 1 [13]. A higher score indicates greater predicted efficacy against the respective bacterial type. These numerical scores help identify peptides that exhibit vigorous overall antimicrobial activity, guiding the selection of candidates with the highest predicted effectiveness for further experimental validation.

In Table 1, peptides 2 (TGWATSGLATFTLHTGSLAPAT) and 3 (TGTATSGLATFTLHTGSLAPAT) were selected for their balanced and high overall antimicrobial activities, with scores of 0.965 and 0.9767, respectively, making them the top candidates for enhanced antibacterial effectiveness. Specifically, peptide 2 demonstrated one of the highest success rates for Gram-positive bacteria, with an antimicrobial activity score of 0.975. Peptide 3 showed strong performance, achieving a 0.6 success rate and an antimicrobial activity score of 0.9767. For Gram-negative bacteria, peptide 2 achieved a high success rate of 0.951, and peptide 3 had a notable rate of 0.736. These values indicate that peptides 2 and 3 possess the highest antimicrobial activity among the other candidates, making them the most promising options for enhanced antibacterial effectiveness. Peptides 2 and 3 were subsequently renamed AtMP2-1 and AtMP2-2, respectively.

The high hydrophobicity ratios (41% and 36%) and consistent total net charge (0.25) across the top peptides suggest that these physicochemical properties are crucial for the antimicrobial efficacy of the selected peptides. These characteristics likely contribute to their ability to interact with bacterial membranes, disrupting cellular integrity and leading to cell death [14].

After the preliminary selection, AtMP2-1 and AtMP2-2 underwent Minimum Inhibitory Concentration (MIC) determination and the Kirby–Bauer Disk Diffusion assay to evaluate their antimicrobial efficacy against various bacterial strains. MIC determination was used to find the lowest concentration of the peptides that effectively inhibits visible bacterial growth, providing insight into their potency [15]. The Kirby–Bauer disk diffusion assay further assessed the antimicrobial activity by measuring the zone of inhibition around the peptide-impregnated Disks placed on agar plates inoculated with bacteria, helping to visualize the peptides’ efficacy in preventing bacterial growth across different species [15].

### 2.2. Antimicrobial Activity

#### 2.2.1. Minimum Inhibitory Concentration Determination

The minimum inhibitory concentration was determined using a 96-well microtiter plate format.

Broth microdilution assays were used to assess the antimicrobial activity of AtMP2, AtMP2-1, and AtMP2-2. According to CLSI definitions, the minimum inhibitory concentration (MIC) is the lowest concentration that completely inhibits visible growth after overnight incubation. Within the tested range (1.25–20 µg/mL), none of the peptides achieved complete inhibition of bacterial growth. Therefore, the actual MIC values were not reached for AtMP2-1 or AtMP2-2. Instead, both peptides demonstrated partial, dose-associated suppression of growth, most notably against *B. cereus*. For *E. coli* and *P. aeruginosa*, inhibition was modest and non-monotonic across concentrations, while *B. subtilis* showed partial suppression but not complete inhibition. These results indicate that the actual MIC values for AtMP2-1 and AtMP2-2 are greater than 20 µg/mL, and the current assays reflect growth suppression rather than formal MIC endpoints.

Replicate variability was low across all organisms. For *E. coli*, SD ranged from 0.007 to 0.074; for *B. subtilis*, from 0.007 to 0.063; for *P. aeruginosa*, from 0.006 to 0.024; and for *B. cereus*, from 0.010 to 0.065.

**Figure 1:**
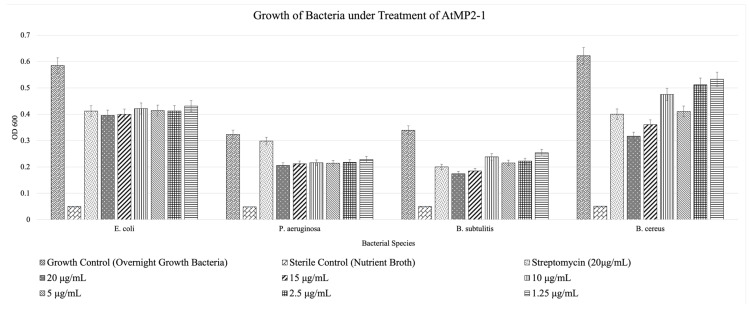
The minimum inhibitory concentration (MIC) of the modified peptide AtMP2-1 was determined using the broth dilution method against various bacteria.

**Figure 2:**
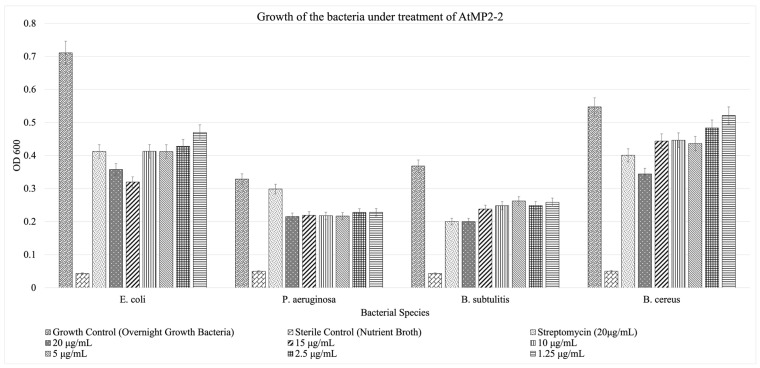
Representing the minimum inhibitory concentration (MIC) of the modified peptide AtMP2-2 measured by the broth dilution method against different bacteria.

Figure 1 and Figure 2 show that while bacterial growth is consistently present across all tested peptide concentrations, there is a notable inhibition compared to the growth control, which contains bacteria only. This suggests that although the bacteria can still grow in the presence of the peptides, their growth is significantly hampered, demonstrating measurable antimicrobial activity.

Across organisms, both AtMP2-1 and AtMP2-2 suppressed growth more effectively than AtMP2. While some organism–peptide pairs exhibited non-monotonic responses across the concentration series, the overall trend indicated dose-associated inhibition, with suppression most apparent for *B. cereus.* Even at the lowest peptide concentrations tested, inhibitory effects were detectable compared with the no-peptide control.

Including streptomycin as a positive control provided a benchmark for evaluating the efficacy of the peptides. Streptomycin demonstrated robust bacterial growth inhibition at 20 µg/mL across all tested strains, serving as a reference point for comparing the activity of the AtMP2-derived peptides.

These findings align with previous studies on antimicrobial peptides (AMPs), which have also reported inhibitory, but not always complete, growth suppression within the tested ranges. For example, Oyama et al. (2022) demonstrated inhibition of Methicillin-Resistant *Staphylococcus aureus* (MRSA) and Gram-negative bacteria by HG2 and HG4 AMPs [15], while Tincho et al. (2020) reported dose-dependent growth inhibition by AMPs against both Gram-positive and Gram-negative species [16].

These results emphasize the potential utility of AtMP-derived peptides as antimicrobial agents. The alignment of these findings with other studies further supports the notion that AtMP-derived peptides could be valuable in combating bacterial infections and antibiotic resistance.

#### 2.2.2. Kirby–Bauer Disk Diffusion

It was found that all three peptides, AtMP2, AtMP2-1, and AtMP2-2, exhibit different antibacterial activities, and these variations are likely due to structural changes that affect how the peptides interact with bacterial membranes. Antimicrobial peptides (AMPs) often function by rupturing microbial membranes through various pathways, including intracellular target interactions, hole formation, and membrane permeabilization [17].

**Figure 3:**
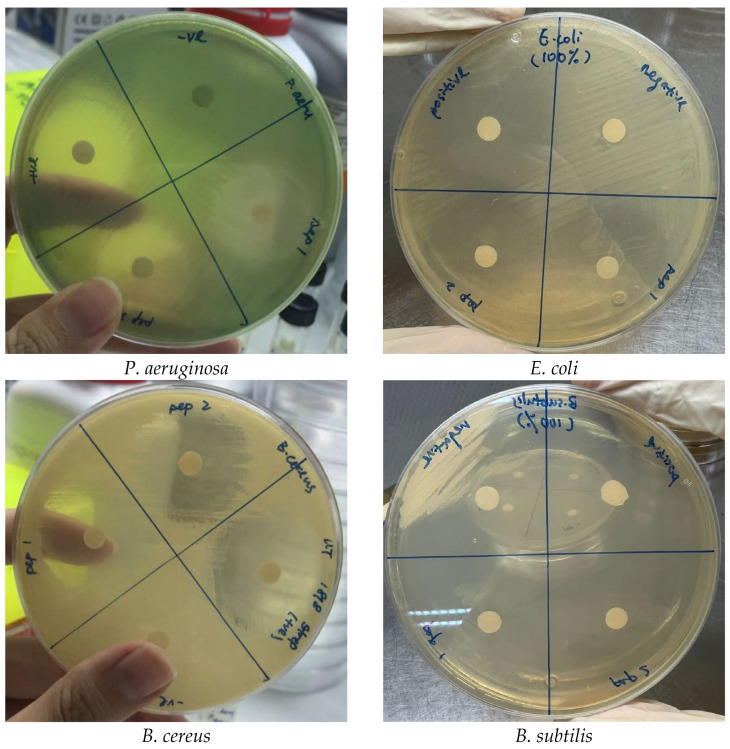
Representative plate photographs of inhibition zones.

The disk diffusion method was employed to assess the antimicrobial activity of AtMP2-1 and AtMP2-2, which were modified from AtMP2, against Gram-positive and Gram-negative bacteria (*E. coli*, *P. aeruginosa*, *B. subtilis* and *B. cereus*). Streptomycin served as a positive control, and the original peptide AtMP2 was used as a reference. Representative plate photographs of the inhibition zones are provided in Figure 3 to illustrate the clearing zones observed. Both modified peptides, AtMP2-1 and AtMP2-2, exhibited larger inhibition zones than the parental AtMP2. For example, both peptides produced inhibition zones exceeding 50 mm against *B. subtilis*. The standard deviation across all tests was approximately 1.15 ± 0.58 mm, reflecting minimal variability in inhibition zone diameters across different bacterial strains.

The results from independent *t*-tests for inhibition zones showed statistically significant differences compared to AtMP2: *E. coli* (AtMP2-1, *p* = 3.34 × 10^−5^; AtMP2-2, *p* = 6.73 × 10^−5^), *B. subtilis* (AtMP2-1 and AtMP2-2, *p* = 3.67 × 10^−17^), *P. aeruginosa* (AtMP2-1 and AtMP2-2, *p* = 0.0132), and *B. cereus* (AtMP2-1, *p* = 1.94 × 10^−5^; AtMP2-2, *p* = 1.52 × 10^−5^). These results confirm that the inhibition zones of AtMP2-1 and AtMP2-2 were significantly greater than those of AtMP2.

The disk diffusion assay further indicated that AtMP2-1 and AtMP2-2 inhibited *P. aeruginosa* and *E. coli* more strongly than the parental AtMP2 peptide. Although the difference between AtMP2-1 and AtMP2-2 was not statistically significant after accounting for variation, both modified peptides consistently outperformed AtMP2 in inhibiting the growth of Gram-negative bacteria. Importantly, inhibition zones in agar reflect a combination of antimicrobial activity and compound diffusion; therefore, comparisons between streptomycin and peptides, or between peptides of different physicochemical properties, should be interpreted cautiously.

As AtMP2 derivatives, AtMP2-1 and AtMP2-2 may have altered physicochemical characteristics such as hydrophobicity, net charge, or secondary structure, which are known to influence AMP activity. These changes could facilitate stronger binding to microbial membranes and subsequent disruption. However, our data do not establish peptide stability or membrane penetration, which remain hypotheses requiring targeted assays to confirm.

AtMP2-1 appeared to suppress Gram-negative bacteria (*P. aeruginosa* and *E. coli*) more effectively, possibly reflecting its structural properties, which favor interaction with their outer membranes. These outer membranes are often difficult to penetrate due to their complex compositions [18]. AtMP2-2 showed greater activity against the Gram-positive *B. cereus*. This trend aligns with general AMP mechanisms described in the literature, where cationic peptides sometimes interact with or penetrate thick Gram-positive cell walls. However, this remains speculative for AtMP2-2 without direct evidence [19].

One reasonable explanation for the observed activity differences lies in the amino acid substitutions introduced into AtMP2-1 and AtMP2-2. While hydrophobicity and net charge changes influence AMP behavior, variations in target-specific interactions—such as affinity for lipid components or cell wall structures unique to each bacterial species—may also contribute to the observed trends [20].

### 2.3. Cytotoxicity Testing—SRB Assay

The SRB assay results demonstrate that AtMP2-1 and AtMP2-2 have low cytotoxicity on HS27 and Vero cells, with cell viability exceeding 80% at all tested concentrations and time intervals. This implies these peptides are relatively safe for application in human (HS27) and animal (Vero) cells, even when exposed to higher concentrations or extended durations.

**Figure 4:**
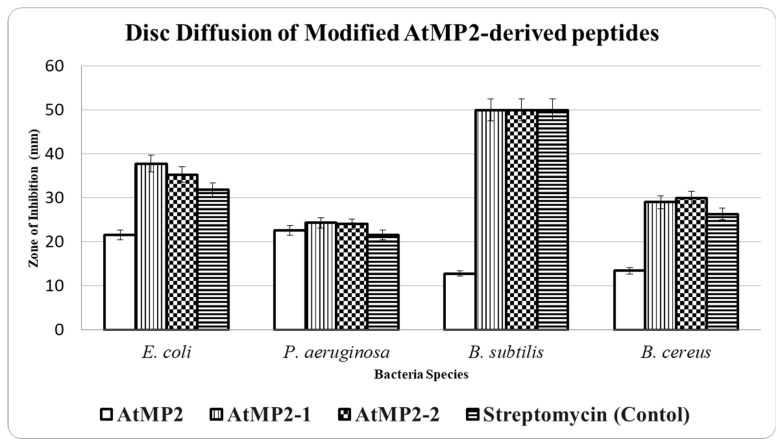
Disk diffusion method for measuring the antibacterial activity of peptides. Zone of inhibition diameters (mean ± SD, n = 3) determined by the Kirby–Bauer disk diffusion method for *E. coli*, *P. aeruginosa*, *B. subtilis*, and *B. cereus*. Streptomycin was used as a positive control, and AtMP2 as the parental reference peptide. Exact *p*-values are reported in the results section.

**Figure 5:**
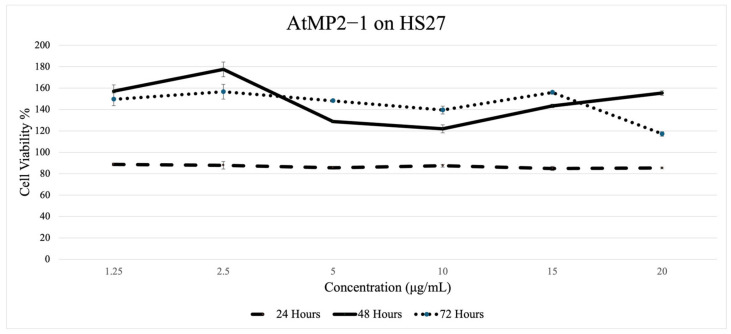
Colorimetric cytotoxicity test of AtMP2-1 on HS-27 Fibroblast Cells through (Sulforhodamine B) Assay.

**Figure 6:**
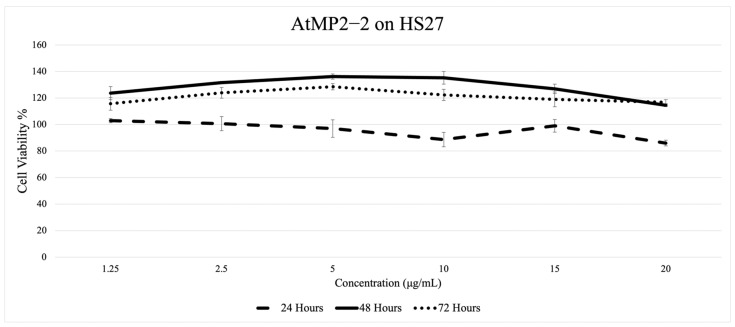
Colorimetric cytotoxicity test of AtMP2-2 on HS-27 Fibroblast Cells through (Sulforhodamine B) Assay.

**Figure 7:**
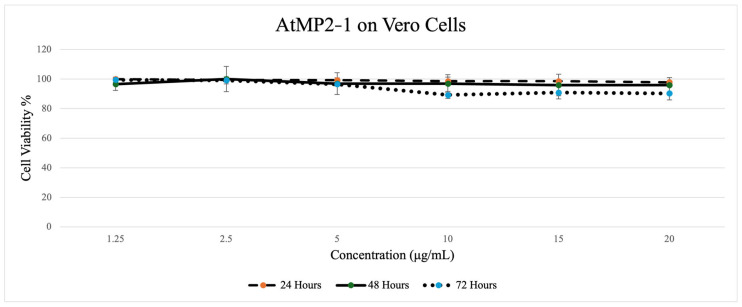
Colorimetric cytotoxicity test of AtMP2-1 on Vero Cells through (Sulforhodamine B) Assay.

Figure 4, Figure 5, Figure 6 and Figure 7 depict the effect of two AtMP2–derived antimicrobial peptides, AtMP2-1 and AtMP2-2, on the cell viability of HS27 and Vero cells over 24, 48, and 72 h at various concentrations (1.25 to 20 µg/mL) over time (24, 48, and 72 h). The SRB assay was used to assess cytotoxicity by measuring cell viability. The study revealed that increasing peptide concentrations significantly decreased the viability of all tested cells.

Figure 5 shows AtMP2-1 on HS27. Cell viability remained consistently high across all concentrations, indicating minimal cytotoxicity after 24 h of incubation. The 48 h trend shows that the cell viability exhibited a slight increase at the lower concentrations before gradually declining, approaching the baseline as the concentration increased. For the 72 h trend, the results were similar to those at the 48 h mark, with a modest elevation in cell viability at lower doses, followed by a gentle decrease at higher concentrations, reaching its lowest point at 20 µg/mL but remaining within the non-cytotoxic range.

Figure 6 shows AtMP2-2 on HS27. At the 24 h trend, cell viability remained relatively stable, staying close to 100% at lower concentrations and decreasing slightly to approximately 96% at higher concentrations. The 48 h trend shows a modest rise at the lower concentrations, reaching just under 100% before gradually declining as the concentration increased, approaching around 95–96% at the highest dose. For the 72 h trend, the pattern was similar to the 48 h results, with a slight elevation around the mid-range concentration, followed by a steady decrease at higher concentrations. However, the decline was less pronounced compared to that observed for AtMP2-1.

Figure 7 shows AtMP2-1 on Vero cells. At the 24 h trend, cell viability remained consistently high, with only slight fluctuations observed across all concentrations and between the treatments. The 48 h trend shows a similarly stable pattern, with viability values remaining near the baseline and showing only minor decreases at the mid- and higher concentrations. For the 72 h trend, the overall pattern aligned with the earlier time points, though a more noticeable reduction in viability was observed at the higher concentrations, with values dipping to around 89–90%, while the lower concentrations remained closer to the baseline.

**Figure 8:**
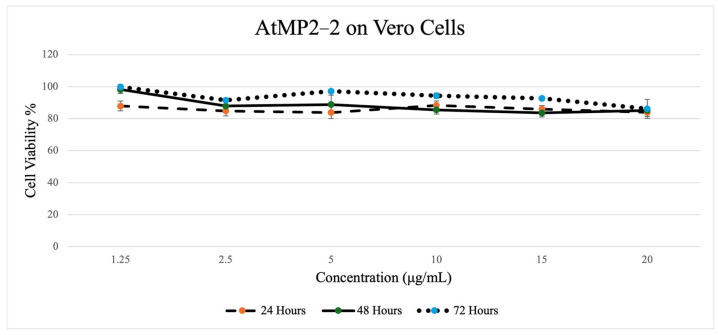
Colorimetric cytotoxicity test of AtMP2-2 on Vero Cells through (Sulforhodamine B) Assay.

At the 24 h mark, cell viability ranged from approximately 88% to 84%, with a slight decrease as the concentration increased, followed by a small rise at 10 µg/mL before declining again toward 20 µg/mL. The 48 h trend exhibited a similar pattern, where cell viability remained close to the baseline at lower concentrations (approximately 98% at 1.25 µg/mL) before gradually decreasing to the mid-80% range at higher concentrations. At 72 h, the overall trend remained consistent with that observed at earlier time points. Cell viability was highest at 1.25 µg/mL (~99.9%), then decreased through the mid-range concentrations, reaching a value of slightly above 86% at 20 µg/mL. Despite the downward trend at higher doses, all values remained within a non-cytotoxic range, as seen in Figure 8.

HS27 cell viability stayed within a safe range at all concentrations and time points, showing only a slight decrease at higher concentrations but never falling below approximately 85%. This suggests that although the peptides exhibit some cytotoxicity at elevated concentrations, they do not cause significant harm to human cells, especially at therapeutic doses. Vero cells demonstrated even greater tolerance to both peptides, with cell viability consistently remaining close to or above 95%, even at the highest concentration of 20 µg/mL. This indicates that the peptides are especially non-toxic to animal cells, making them a safe choice for therapeutic use in animal models.

Previous research on AMPs has highlighted varying levels of cytotoxicity depending on the peptide and target cell type. For instance, a study by Theansungnoen et al. (2016) showed that the AMPs KT2 and RT2, derived from *Crocodylus siamensis* leukocyte extract, had significant cytotoxic effects on human cervical cancer cells, with IC50 values ranging from 28.7 to 53.4 µM [21]. In contrast, the results from the current study demonstrate that AtMP2-1 and AtMP2-2 maintain much higher cell viability levels, with only mild reductions observed even at concentrations up to 20 µg/mL. This suggests that these peptides are safer and less cytotoxic than other AMPs reported in the literature.

AtMP2-1 exhibited cell viability values ranging from approximately 88% to 100% in HS27 cells and from 95% to 100% in Vero cells, even at the highest concentration tested. Similarly, AtMP2-2 maintained cell viability within the 85–100% range in both cell lines, with only a slight reduction observed at 20 µg/mL. The minimal decrease in cell viability across various concentrations and time points underscores their safety and suggests that these peptides are well-tolerated by human and animal cells [22,23]. Maintaining high cell viability across a range of concentrations and exposure times indicates that these peptides can be used therapeutically without causing significant harm to healthy cells, making them promising candidates for further development in antimicrobial therapy, especially in scenarios where low cytotoxicity is crucial.

### 2.4. Bioinformatics (Protein-Peptide Docking Analysis)

Ten *E. coli* proteins were selected as representative targets for the docking study, spanning key bacterial cellular functions, based on their frequent use in antimicrobial peptide docking studies. These included DNA gyrase subunits A and B (GyrA, GyrB) and DNA topoisomerase IV subunit B (ParE), which are involved in DNA replication; the RNA polymerase β-subunit (RpoB) for transcription; SecA for protein translocation; GroEL and DnaK as molecular chaperones; ClpP as an ATP-dependent protease; methionyl-tRNA synthetase (MetG) for protein synthesis; and FtsZ for cell division. It should be noted that some of these proteins (e.g., DnaK and ClpP) are not strictly essential under all growth conditions but were included here as they represent functionally important bacterial pathways that are often explored in peptide–protein interaction studies.

Docking was performed using two web-based tools, ZDOCK and HPEPDOCK. These platforms predict peptide–protein binding conformations and assign docking scores based on predicted binding energies. More negative scores indicate stronger predicted binding affinity; however, these values are relative and do not equate to experimentally measured affinities (e.g., Kd or EC50). Using two independent servers allowed cross-checking results, but the outcomes should be considered hypothesis-generating predictions rather than proof of direct interactions.

While docking suggested possible binding compatibilities of AtMP2-1 and AtMP2-2 with several of these proteins, it is doubtful that the peptides interact with all 10 proteins in vivo. Instead, these findings highlight plausible molecular targets that warrant further investigation. Confirming these interactions will require direct experimental validation using approaches such as surface plasmon resonance (SPR), isothermal titration calorimetry (ITC), microscale thermophoresis (MST), or functional inhibition assays.

The protein targets identified for AtMP2-1 and AtMP2-2 in this study are based on docking simulations and literature comparisons. No experimental data were obtained here to determine binding affinities (Kd/EC50) or to confirm membrane penetration. These results should therefore be regarded as hypotheses, requiring follow-up validation with biophysical and cell-based assays.

Table 2 displays the docking scores, which indicate the binding affinities of the peptides to the target proteins. The docking poses of AtMP2-1 and AtMP2-2 with selected proteins are illustrated.

Table 2 summarizes the docking scores and bond types of two antimicrobial peptides, AtMP2-1 and AtMP2-2, against 10 target proteins involved in the bacterial cell death cycle. The table is divided into two main sections, showing results from two different docking servers, HPEPDOCK and ZDOCK. In the ZDOCK models, the gray shadow represents the predicted binding region, while the peptides are shown in purple. In the HPEPDOCK models, the peptides are displayed in yellow and the proteins in brown.

The docking score indicates the binding affinities between the peptides and the target proteins. Lower (more negative) scores suggest stronger interactions. For example, a docking score of −199.731 for GyrA indicates a strong binding affinity for the AtMP2-1 peptide with one of the proteins. Various scoring functions may yield different docking scores based on how they weigh these factors, offering insight into the potential binding strength and guiding candidate selection for further experimental validation [24].

**Figure 9:**
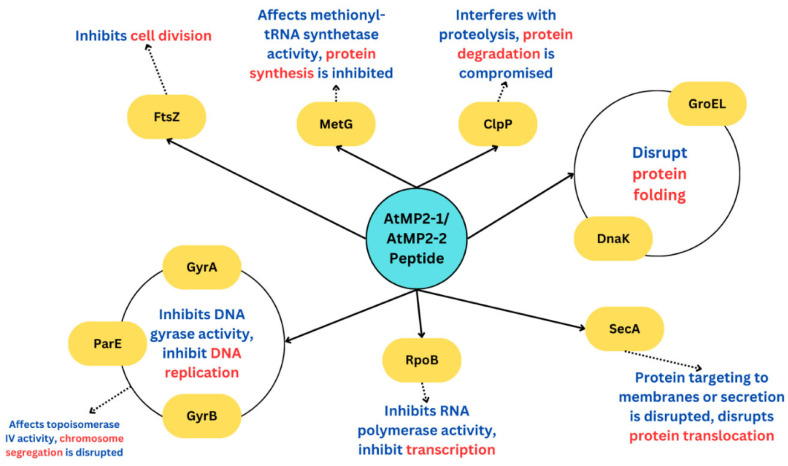
Hypothetical Pathway of AtMP2-1 and AtMP2-2 Peptides Interaction with Cellular Proteins Leading to Bacterial Cell Death.

The sequential interactions between the peptide and cellular proteins, which ultimately lead to cell death, are depicted in the illustrated pathway diagram (Figure 9). The relationships were hypothesized based on the established roles of these proteins in vital physiological functions, including transcription, DNA replication, protein translocation, folding, degradation, synthesis, and cell division.

Table 3 provides a detailed summary of various bacterial genes and proteins, suggesting how peptides AtMP2-1 and AtMP2-2 disrupt their functions, potentially leading to bacterial cell death.

Molecular docking was used to generate a hypothesis regarding the possible bacterial protein targets of AtMP2-1 and AtMP2-2. The results indicated strong predicted affinities for essential proteins such as GyrA, RpoB, SecA, ClpP, and MetG. These proteins are fundamental to bacterial survival, and several previously studied antimicrobial peptides have been shown to interact with them [25,26,27,28,29,30,31].

#### 2.4.1. Inhibition and DNA Replication

Targeting the crucial components of the DNA replication machinery, starting with the DNA gyrase, the peptide starts its cytotoxic route. DNA gyrase, which comprises the subunits GyrA and GyrB, is necessary to release the torsional strain that develops before the replication fork during DNA replication.

Docking predictions suggested that AtMP2-1 and AtMP2-2 could interact with bacterial targets such as GyrA and GyrB, proteins essential for DNA gyrase activity [32]. Such interactions have been described for other antimicrobial peptides in the literature and may partly explain the observed antibacterial effects of AtMP2 variants. However, these mechanisms remain hypothetical for the peptides studied here and require further validation in experimental assays.

Besides DNA gyrase, the peptide interacts with ParE, a subunit of topoisomerase IV [33]. After DNA replication, tangled daughter chromosomes must be decatenated or untangled; topoisomerase IV is essential for this process. The peptide disrupts chromosome segregation, which is necessary for proper cell division, by binding to ParE. Additionally, it significantly inhibits DNA replication by targeting and blocking the activity of both DNA gyrase and topoisomerase IV. This causes increased DNA damage, chromosome missegregation, and cell death [34]. This multifunctional strategy guarantees a thorough disruption of DNA replication and cell cycle development, efficiently initiating the cytotoxic effects required to eradicate bacteria.

The possibility of inhibition in the above is allosteric inhibition or competitive inhibition. This is because GyrA, GyrB, and ParE have multiple potential binding sites beyond the active site. If the peptide binds to one of these allosteric sites, it could cause a change in the protein’s conformation that indirectly affects the active site or other functional areas, thereby inhibiting the protein’s activity [34]. Allosteric inhibition occurs when a peptide binds to a protein region other than the active site, causing a conformational change that alters the protein’s structure and function. This change can impact the protein’s overall structure and its functional domains [35].

#### 2.4.2. Suppression of Transcription

The suppression of transcription is a critical mechanism through which antimicrobial peptides exert their lethal effects on bacterial cells. AtMP2-1 or AtMP2-2 first attaches itself to the RNA polymerase component RpoB. The enzyme RNA polymerase creates mRNA from DNA templates, which is necessary for expressing genes involved in various cellular processes and ensuring cellular survival [36]. The peptides are predicted to inhibit RNA polymerase activity by binding to the RpoB subunit, potentially interfering with transcription.

The disruption of the transcriptional machinery resulting from this inhibition stops the production of mRNA. Translating these signals into functional proteins is stopped without mRNA synthesis [37]. Critical cellular functions such as metabolism, structural integrity, and response to external stressors are impacted when newly synthesized proteins are absent [38]. The peptide’s stress and damage are further increased by the disruption of gene expression, which also hinders the production of regulatory proteins that manage different cell pathways [38]. The failure of cellular repair and maintenance mechanisms ultimately results from the inability to produce the necessary proteins, which contributes to the accumulation of cellular damage [39]. The bacterial cell eventually experiences a stressful situation due to disturbed transcriptional activity, resulting in protein shortage, which drives the bacterial cell towards apoptosis or programmed cell death. This complete suppression of transcription highlights the peptide’s vigorous antibacterial activity, as it methodically destroys the cellular infrastructure required for bacterial growth.

#### 2.4.3. Disruption of Protein Translocation and Folding

The peptide interferes with two essential processes for cellular function: protein folding and translocation. Based on protein-peptide docking of AtMP2-1 and AtMP2-2, these disruptions can lead to protein misfolding or hinder the proper movement of proteins across membranes, ultimately impairing the bacteria’s ability to maintain homeostasis and execute vital functions [40]. As a result, these peptides could induce cellular stress and contribute to eventual cell death, supporting their potential as effective antimicrobial agents.

It targets SecA, a crucial component of the Sec translocon that transports newly synthesized proteins across the cell membrane [27]. SecA’s inhibition by the peptide prevents proteins from reaching their destined locations, accumulating proteins in the cytoplasm, and triggering cellular stress.

The peptides also interact with chaperone proteins DnaK and GroEL, which are necessary for protein folding. DnaK and GroEL help young proteins fold correctly to take on their functional conformations [28].

#### 2.4.4. Inhibition of Protein Homeostasis

Through two different mechanisms, the suppression of protein production and the prevention of protein degradation, the AtMP2-1 and AtMP2-2 peptides significantly impact protein homeostasis.

First, AtMP2-1 or AtMP2-2 attaches itself to MetG, an essential enzyme involved in producing methionine-tRNA, the first step in protein synthesis [41]. Both peptides efficiently inhibit the translation process by targeting MetG, resulting in a marked decrease in the synthesis of new proteins essential for various cellular processes and survival. The cell’s capacity to maintain normal physiological functions is compromised by this suppression, resulting in a state of protein deficiency.

At the same time, the peptide targets ClpP, an essential protease that breaks down and recycles broken or misfolded proteins. ClpP is necessary for controlling protein quality because it ensures that abnormal proteins are effectively eliminated, preventing harmful buildup inside the cell [29]. When the peptide and ClpP bind, the proteolytic activity of ClpP is inhibited, resulting in a buildup of broken and misfolded proteins. Proteotoxic stress is the outcome of this accumulation, whereby the presence of malfunctioning proteins overwhelms the cell’s machinery. The delicate balance of protein homeostasis is disrupted by the combined effects of these processes, including the inhibition of defective protein breakdown and the suppression of new protein synthesis, ultimately leading to cellular malfunction and death. This complex disruption underscores the peptide’s comprehensive approach to targeting vital cellular processes and achieving antimicrobial effects.

#### 2.4.5. Interruption of Cell Division

A crucial part of AtMP2-1 and AtMP2-2’s cytotoxic route involves a specific interaction with the bacterial protein FtsZ, interrupting the cell cycle.

FtsZ, a tubulin-like protein, polymerizes to form the Z-ring at the site of future cell division, playing a vital role in bacterial cytokinesis. This Z-ring is essential for septum formation and serves as a scaffold for recruiting additional proteins required for cell division [31]. This Z-ring cannot be correctly assembled when the antimicrobial peptide attaches to the protofilaments formed by FtsZ monomers. This interaction prevents the development of a working division apparatus, which prevents the required division proteins from being recruited. Cytokinesis is blocked because of the cell’s inability to generate the septum necessary for binary fission. The bacteria proliferate without dividing, becoming filamentous and multinucleated due to their failure to complete cell division. This causes cellular stress to accumulate, ultimately triggering pathways for programmed cell death [31]. The antimicrobial peptide significantly contributes to its overall bactericidal activity by effectively halting the proliferation of bacterial cells by targeting FtsZ and disrupting the fundamental process of cell division.

#### 2.4.6. Limitations and Potential Impact

As most of the experiments conducted were in vitro, it may not be enough to fully replicate the complexities of in vivo conditions, including factors such as peptide stability in the bloodstream, immune responses, and bioavailability. The lack of in vivo testing means that the therapeutic potential of these peptides requires validation through animal models or clinical studies. Additionally, while identifying significant interactions, the docking studies were limited to a select few bacterial proteins. This narrow scope may have overlooked other potential targets and mechanisms of action. Another limitation is the unassessed stability of AtMP2-1 and AtMP2-2 in protease-rich environments. Proteolytic degradation could limit their effectiveness in physiological conditions, reducing their therapeutic viability. Moreover, the study tested the peptides on only four bacterial strains (*E. coli*, *P. aeruginosa*, *B. subtilis*, and *B. cereus*), leaving the activity spectrum untested against a broader range of Gram-positive and Gram-negative bacteria, including multidrug-resistant strains.

The potential impacts of these limitations should also be examined. The in vitro approach may overstate the efficacy and safety of peptides, as physiological conditions may introduce variables that reduce their activity or increase their toxicity. Limited docking investigations may suggest that uncharacterized interactions or off-target effects could impact the safety and efficacy of these peptides in therapeutic applications. Furthermore, without stability assessments, the peptides may degrade rapidly in vivo, severely limiting their therapeutic window and overall efficiency.

The initial in silico optimization of AtMP2 variants focused on increasing hydrophobicity and net charge, criteria commonly associated with membrane-active AMPs. However, the exact mechanism of AtMP2 is unknown, and our docking predictions suggest that intracellular targets may also play a role in its function. Thus, while hydrophobicity and charge-based selection provided valid leads, this approach remains limited without mechanistic validation.

To address these limitations, future research should prioritize several key areas. In vivo studies are crucial for assessing the pharmacokinetics, pharmacodynamics, and immune responses triggered by the peptides. Additionally, future optimization efforts should incorporate additional structural and mechanistic criteria once the mode of action of AtMP2 is clarified. Expanding the range of bacterial strains tested, with a primary focus on multidrug-resistant pathogens, will provide a more in-depth understanding of the peptides’ broad-spectrum utility. Finally, investigating combination therapies, where AtMP-derived peptides are used in conjunction with conventional antibiotics, may enhance their effectiveness and help mitigate the development of resistance. Addressing these limitations will enable further validation of the therapeutic potential of AtMP2-1 and AtMP2-2, advancing their development as next-generation antimicrobial agents.

## 3. Materials and Methods

### 3.1. Peptide Selection

AtMP2 (TGIATSGLATFTLHTGSLAPAT), derived from the epidermal mucus of *A. testudineus*, was modified in silico by changing a single amino acid and generating 440 modified peptides [10]. The Antimicrobial Peptide Database (APD3) “https://aps.unmc.edu/prediction (accessed on 20 February 2023)” was initially utilized to predict peptide properties, including structure, function, hydrophobicity, and net charge [42,43]. A single-site saturation mutagenesis approach was employed to generate peptide variants, wherein each amino acid position in the AtMP2 sequence was individually substituted with alternative amino acids. This approach resulted in 440 unique peptide sequences. The top 20 peptides that meet the criteria for high hydrophobicity and a positive net charge, as determined by comparison in Excel, were chosen, as these properties are associated with better antimicrobial activity.

The Collection of Antimicrobial Peptides (CAMP) database (http://www.camp3.bicnirrh.res.in/predict/) was then utilized to distinguish between antimicrobial and non-antimicrobial peptides, focusing on total hydrophobic ratio, stability, and net charge [13]. Peptides that demonstrate antimicrobial properties will be selected.

Finally, the AMPFun database (http://fdblab.csie.ncu.edu.tw/AMPfun/index.html) was utilized to predict the effectiveness of peptides against Gram-positive and Gram-negative microbes, as well as their potential anticancer properties [44,45,46]. The top 20 peptides were selected after being evaluated on these three websites.

After evaluating these three databases, only the top 2 peptides with a balanced and high overall antimicrobial activity were selected for synthesis and further experimental validation. These peptides were tested to confirm their enhanced antibacterial efficacy and safety for potential therapeutic applications.

### 3.2. Peptide Synthesis

To test enhanced antibacterial efficacy and safety, the selected peptides were ordered and synthesized by Genscript Singapore in powdered form. Upon arrival, the synthesized peptides were activated using ultrapure water, as recommended by the manufacturer.3.3. Antimicrobial Activity

### 3.3. Antimicrobial Activity

#### 3.3.1. Kirby–Bauer Disk Diffusion

The disk diffusion method is widely used for assessing the antimicrobial activity of various compounds [47]. Two of the top 20 modified peptides of the epidermal mucus extract derived from *A. testudineus* were tested for antibacterial efficacy on four bacterial strains. The bacterial strains used for antimicrobial testing were *Escherichia coli* (ATCC 25922), *Pseudomonas aeruginosa* (ATCC 27853), *Bacillus subtilis* (ATCC 6051), and *Bacillus cereus* (ATCC 14579). All strains were obtained from the American Type Culture Collection (ATCC) and maintained according to ATCC-recommended protocols. Bacteria were cultured in nutrient broth and subcultured onto Mueller-Hinton agar for susceptibility testing. These microbes are selected because they consist of Gram-positive and Gram-negative, clinically essential bacteria in human infection [47].

The Kirby–Bauer disk diffusion method was utilized to evaluate the antimicrobial activity of the modified peptide against microbial strains. The growth and turbidity of each culture were measured against the 0.5 McFarland Turbidity Standard and corrected using sterile distilled water to match the standard’s turbidity [47]. A tenfold serial dilution was applied to the cultures, and then 50 µL of the diluted sample was added to an MH agar plate [48]. The bacterial culture was evenly lawned on the agar surface using a sterile cotton swab [47]. Before proceeding to the next step, the lawned plates were allowed to dry for 5–10 min.

A total of 20 µL (20 µg/mL) of AtMP2-1 or AtMP2-2 peptide, diluted using autoclaved deionized water, was applied to a 6 mm blank antibiotic disk [49]. The antibiotic disks were placed on the surface of the agar and gently pressed to ensure they were firmly in place [47]. The standard antibiotic disk (20 µg/mL streptomycin) was used as the positive control, and the non-activated antibiotic disk served as the negative control. A total of 4 disks were placed on a single plate in each of the designed quadrants. The inhibitory zones, measured to the nearest millimeter (mm), were calculated using three different tests.

AtMP2-1 and AtMP2-2, which exhibit antimicrobial activity, were tested to determine their minimum inhibitory concentration (MIC), defined as the lowest concentration of the peptide required to inhibit the growth of pathogens [50].

#### 3.3.2. MIC-Range Growth Inhibition Assay

The microdilution method was used to determine the minimum inhibitory concentration of the modified peptide. A spectrophotometer measured the turbidity of the culture, which was adjusted to the McFarland turbidity standard—an OD reading of 0.5 (1 × 10^8^ CFU/mL)—using sterile distilled water to achieve the required turbidity.

On the other hand, different dilutions of AtMP2-1 and AtMP2-2 were made using a two-fold serial dilution. 96-well microdilution plates were prepared. The concentration range must encompass the anticipated MIC of the tested microorganism.

Two controls were used in this section: (1) the growth control (positive control), consisting of overnight-grown bacteria in nutrient broth, and (2) the sterile control (negative control), containing only sterile nutrient broth. Incubation was carried out at 37 °C for 18 h, and turbidity was determined using a plate reader. The MIC of each modified peptide and different combinations of microbes was recorded and analyzed [50].

### 3.4. Cytotoxicity Testing (SRB Assay)

A sulforhodamine B (SRB) assay was used to test the cytotoxicity of the modified peptides. The human fibroblast cell line HS-27 (ATCC CRL-1634) and African green monkey kidney cell line Vero (ATCC CCL-81) were obtained from the American Type Culture Collection (ATCC). Cells were cultured in appropriate media (fibroblast medium for HS-27; DMEM for Vero) and incubated under standard conditions (37 °C, 5% CO_2_). This assay is a colorimetric assay that evaluates the viability and growth of cells. SRB dye, which binds to cellular proteins and may be quantified to assess the presence of live cells, is used in the experiment to fix and stain cells.

#### 3.4.1. Treatment Solution Preparation

Two-fold serial dilution was carried out using deionized water as the solvent in this section. 10 mL of the original samples were added to the 15 mL centrifuge tube [51,52].

#### 3.4.2. Cell Preparation

For cell preparation, the medium was first removed from the T-75 flask. To wash the cells, 3 mL of sterilized PBS is used. To wash the cells, 3 mL of sterilized PBS was added to the T-75 flask. Then, 2 mL of 0.25% (*w*/*v*) trypsin was added and gently shaken to cover the cell-growth surface evenly [14]. Incubate the flask for 10 min at 37 °C in an incubator. The cells were detached from the surface of the flask during the incubation period. After 10 min, the cells were transferred with trypsin into a 15 mL centrifuge tube and adjusted to 8 mL with sterile PBS. The tube was centrifuged for 10 min at 2200 rpm. After centrifugation, the supernatant was discarded, and fresh medium was added to resuspend the cells. 100 µL of the cell suspension and 100 µL of 0.4% (*w*/*v*) trypan blue solution were transferred into a microcentrifuge tube, forming a 1:1 mixture and resuspending in the tube [14]. The mixture was transferred to a hemacytometer chamber for cell counting under a microscope to determine the cell viability. Only the cells that exclude trypan blue (appearing transparent) are considered viable under the microscope [14].

The cell concentration to be transferred into the 96-well plate will be adjusted with the medium. An example of the calculation can be referred to below:

Viable cells under the microscope: Z cells/square

Total concentration: Z cells/square × 16 squares

Total concentration needed: 1 × 10^5^ cells/well

100 µL/well = 100 µL × 96 wells × 3 plates

= 28.8 mL (Round to 30 mL)

M_1_V_1_ = M_2_V_2_

M_1_ = Z

M_2_ = 1 × 10^5^

V_2_ = 30 mL (1)

The concentration was mixed in a reservoir (basin) to facilitate efficient use of the multichannel pipette for pipetting into the 96-well plate. Subsequently, 100 µL of the mixture was seeded into each well. The plate was incubated for 24 h to allow the cells to adhere to the surface.

#### 3.4.3. Treatment Exposure

After 24 h of incubation, 50 µL of the treatment solution was added to each well [14]. After adding the treatment, the plates were incubated with 5% CO_2_ for 48 h [14].

#### 3.4.4. Cell Fixation and Staining

25 μL of astonishing, 50% (*w*/*v*) trichloroacetic acid (TCA) was added to each well and incubated at 4 °C for one h. The plates were rinsed four times under gently running tap water, and any excess water was removed using a paper towel before air drying. After drying, 50 μL of 0.04% (*w*/*v*) SRB solution was added to each well. To remove the unbound dye, the plates were immediately rinsed four times with 1% (vol/vol) acetic acid and left at room temperature for one hour. Allow the 96-well plate to air dry at room temperature [14]. To dissolve the protein-bound dye, 50 to 100 μL of 10 mM Tris base solution (pH 10.5) was added to each well. The plates were shaken on an orbital shaker for 10 min. A microplate reader measured the absorbance at 510 nm [14].

### 3.5. Protein Docking Simulation

Molecular docking was conducted to explore the potential mechanisms of action of the selected peptides at a molecular level. The aim was to predict how AtMP2-1 and AtMP2-2 interact with bacterial proteins involved in essential cellular functions such as DNA replication, transcription, protein synthesis, and cell division. By identifying favorable binding interactions, docking helps validate the antimicrobial targets of the peptides and provides insights into their bactericidal pathways. To achieve this, HPEPDock “http://huanglab.phys.hust.edu.cn/hpepdock/ (accessed on 21 June 2023)” and ZDOCK “https://zdock.wenglab.org/ (accessed on 21 June 2023)” were utilized to investigate the binding affinities and interactions of AtMP-derived peptides, specifically AtMP2-1 and AtMP2-2, with target proteins involved in bacterial cell death. These target proteins included DNA gyrase subunit A (GyrA), RNA polymerase β-subunit (RpoB), preprotein translocase ATPase (SecA), DNA gyrase subunit B (GyrB), chaperonin GroEL, DNA topoisomerase IV subunit B (ParE), chaperone protein DnaK, ATP-dependent Clp protease proteolytic subunit (ClpP), methionyl-tRNA synthetase (MetG), and cell division protein FtsZ. Table S1 (Supplementary Materials) lists the Protein Data Bank (PDB) IDs of the docking structures. The bacterial target proteins selected for docking were chosen based on their essential roles in critical cellular processes, including DNA replication, transcription, protein folding, protein degradation, and cell division. These proteins represent conserved, functionally indispensable components of bacterial survival mechanisms and are commonly investigated as targets for antimicrobial drugs. Targeting such essential proteins increases the likelihood that the peptides can exert bactericidal effects by interfering with fundamental biological pathways. This protein selection strategy aligns to identify peptides that function through multi-target disruption, potentially reducing the risk of resistance development.

To run the docking simulations, the PDB files of the target proteins must be obtained first. For this, the RCSB Protein Data Bank “https://www.rcsb.org/?ref=nav_home (accessed on 22 June 2023)” was accessed. Each protein was searched for, selected, and downloaded in PDB format, serving as the docking program’s input. The HPEPDock tool simulated the binding interaction between each peptide and protein target. The HPEPDock and ZDOCK algorithms evaluate potential binding modes and affinities based on the available conformational space, utilizing energy minimization and scoring functions to identify the most stable complexes. After obtaining the results from HPEPDock and ZDOCK, Discovery Studio 2021 was used to gain further insights into the binding interactions, enabling detailed visualization and analysis of how the peptides fit into the active sites of the target proteins.

Higher negative scores indicated stronger binding affinities, suggesting more favorable interactions. These scores provided a preliminary understanding of how effectively each peptide could bind to its target protein [53].

The resulting data provided insight into the molecular mechanisms by which these peptides exert their antimicrobial effects. HPEPDOCK provided crucial information on docking scores, quantifying the strength of the peptide-protein interactions. The detailed mechanisms of these interactions will be discussed, and key findings will be illustrated to provide a clear understanding of how AtMP2-1 and AtMP2-2 peptides interact with the target proteins to induce bacterial cell death.

### 3.6. Statistical Analysis

All data analyses were performed using Microsoft Excel (Microsoft Corp., USA). For antimicrobial assays (MIC and disk diffusion), each condition was measured using eight technical replicates (A–H). Mean optical density (OD_600_) values and standard deviations (SD) were calculated for each peptide concentration. Error bars representing ±SD were incorporated into Figure 1 and Figure 2 to visualize replicate variability.

For disk diffusion data, independent *t*-tests were used to compare inhibition zones between the parental peptide (AtMP2) and modified peptides (AtMP2-1 and AtMP2-2), with *p*-values reported in the Results section.

Cytotoxicity data from the SRB assay were expressed as mean ± SD from technical replicates. No imputation or outlier removal was performed. Statistical significance was defined as *p* < 0.05.

## 4. Conclusions

Two modified peptides, AtMP2-1 and AtMP2-2, were successfully generated from the native AtMP2 sequence using systematic single-amino-acid substitution and in silico screening. Both peptides demonstrated improved antimicrobial activity against representative Gram-positive and Gram-negative bacteria, while exhibiting low cytotoxicity toward HS-27 and Vero cells at tested concentrations. Docking analysis suggested possible interactions with key bacterial proteins, offering preliminary insight into their potential mechanisms of action.

Overall, AtMP2-1 and AtMP2-2 show promise as candidates for further development as antimicrobial agents. Future studies should validate their molecular targets, assess peptide stability, and evaluate their efficacy in vivo to support their potential therapeutic application.

## Figures and Tables

**Table 1:** The top 20 modified peptides that contain antibacterial properties and are effective towards Gram-positive and Gram-negative bacteria.

No	AMPs Sequence	AMPFun				APD3	
		Antimicrobial Activity	Anticancer Activity	Gram+	Gram−	Total Hydrophobic Ratio %	Total Net Charge
1	TGIATSGLFTFTLHTGSLAPAT	YES 0.9607	YES 0.6884	YES 0.6333	YES 0.7283	41%	0.25
2	TGWATSGLATFTLHTGSLAPAT	YES 0.965	YES 0.6102	YES 0.6	YES 0.7363	41%	0.25
3	TGTATSGLATFTLHTGSLAPAT	YES 0.9767	YES 0.5691	YES 0.575	YES 0.6961	36%	0.25
4	TGTATSGLATFTLHTGSLAPAT	YES 0.9607	YES 0.5441	YES 0.5583	YES 0.6913	36%	0.25
5	TGLATSGLATFTLHTGSLAPAT	YES 0.965	YES 0.5231	YES 0.6167	YES 0.7078	41%	0.25
6	TGIATSGLATFTLHTGSLAIAT	YES 0.9719	YES 0.5218	YES 0.7417	YES 0.7798	45%	0.25
7	TGMATSGLATFTLHTGSLAPAT	YES 0.956	YES 0.5191	YES 0.5583	YES 0.6837	41%	0.25
8	TGIATSGLATFTLHTISLAPAT	YES 0.9526	YES 0.5151	YES 0.65	YES 0.7754	45%	0.25
9	TGIATSGLATFTLHTGSLAPIT	YES 0.9638	YES 0.5113	YES 0.6333	YES 0.792	41%	0.25
10	TGIATSGLATFTLHTGSLIPAT	YES 0.9583	YES 0.5113	YES 0.6583	YES 0.7509	41%	0.15
11	TGIATSGLATFTLHTGSLAFAT	YES 0.9719	YES 0.5001	YES 0.725	YES 0.7497	45%	0.25
12	TGIATSGLATITLHTGSLAPAT	YES 0.9538	YES 0.4991	YES 0.65	YES 0.7514	41%	0.25
13	TGIATSGLATFTLHTGWLAPAT	YES 0.9626	YES 0.4953	YES 0.6583	YES 0.7774	45%	0.25
14	TGIATSGLATFTLHTLSLAPAT	YES 0.9526	YES 0.4953	YES 0.65	YES 0.762	45%	0.25
15	TGIATSGLATFTLHTMSLAPAT	YES 0.9396	YES 0.4953	YES 0.625	YES 0.7301	45%	0.25
16	TGIATSGLATFTLHTTSLAPAT	YES 0.9626	YES 0.4953	YES 0.6583	YES 0.7288	41%	0.25
17	TGIATSGLATFTLHTVSLAPAT	YES 0.9479	YES 0.4953	YES 0.65	YES 0.7538	45%	0.25
18	TGIATSGLATFTLHTGSLAPAI	YES 0.983	YES 0.4952	YES 0.6833	YES 0.7967	45%	0.25
19	TGIITSGLATFTLHTGSLAPAT	YES 0.9504	YES 0.4918	YES 0.5833	YES 0.7409	41%	0.25
20	TGIATSGLATFTLHTGSLACAT	YES 0.9719	YES 0.4911	YES 0.7417	YES 0.768	45%	0.25

**Table 2:** Docking scores and bond types of AtMP2-1 and AtMP2-2 peptides against proteins involved in the bacterial cell death cycle (Full resolution images are provided in Table S2.

Protein	ZDOCK Server		HPEPDOCK Server		Docking Score & Bond Type	Docking Score & Bond Type
	AtMP2-1 (TGTATSGLATFTLHTGSLAPAT)	AtMP2-2 (TGWATSGLATFTLHTGSLAPAT)	AtMP2-1 (TGTATSGLATFTLHTGSLAPAT)	AtMP2-2 (TGWATSGLATFTLHTGSLAPAT)	AtMP2-1 Retrieved on (25 September 2024)	AtMP2-2 Retrieved on (25 September 2024)
GyrA	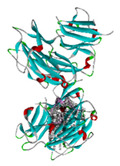 Link Retrieved on (25 September 2024)	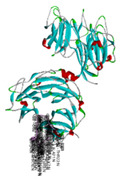 Link Retrieved on (25 September 2024)	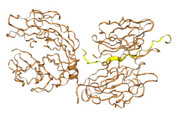 Link Retrieved on (25 September 2024)	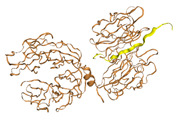 Link Retrieved on (26 September 2024)	−199.731 Single	−209.673 Single
GyrB	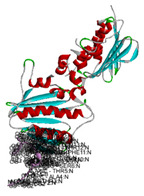 Link Retrieved on (25 September 2024)	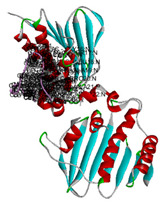 Link Retrieved on (25 September 2024)	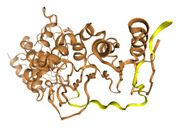 Link Retrieved on (25 September 2024)	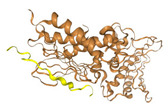 Link Retrieved on (26 September 2024)	−215.102 Single	−204.34 Single
RpoB	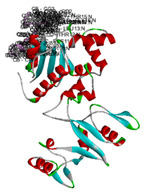 Link Retrieved on (25 September 2024)	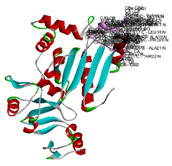 Link Retrieved on (25 September 2024)	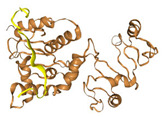 Link Retrieved on (25 September 2024)	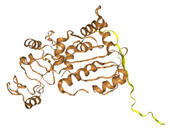 Link Retrieved on (26 September 2024)	−184.743 Single	−182.555 Single
SecA	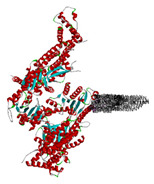 Link Retrieved on (25 September 2024)	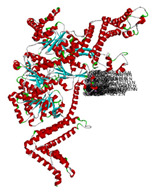 Link Retrieved on (25 September 2024)	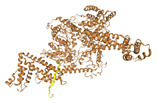 Link Retrieved on (25 September 2024)	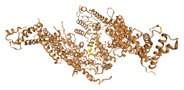 Link Retrieved on (26 September 2024)	−216.083 Single	−230.044 Single
GroEL	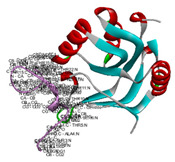 Link Retrieved on (25 September 2024)	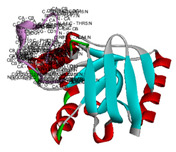 Link Retrieved on (25 September 2024)	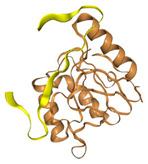 Link Retrieved on (25 September 2024)	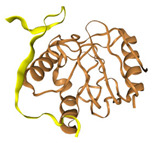 Link Retrieved on (26 September 2024)	−184.075 Single	−186.028 Single
ParE	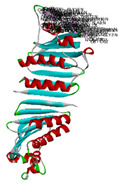 Link Retrieved on (25 September 2024)	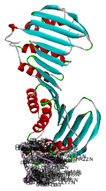 Link Retrieved on (25 September 2024)	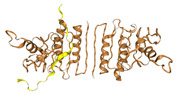 Link Retrieved on (25 September 2024)	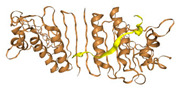 Link Retrieved on (26 September 2024)	−201.482 Single	−195.991 Single
DnaK	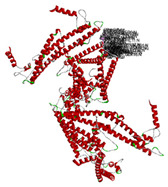 Link Retrieved on (25 September 2024)	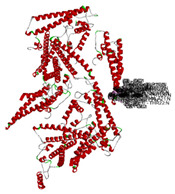 Link Retrieved on (25 September 2024)	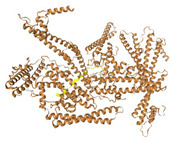 Link Retrieved on (26 September 2024)	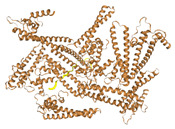 Link Retrieved on (26 September 2024)	−211.956 Single	−211.956 Single
ClpP	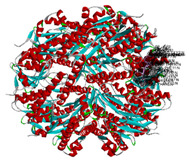 Link Retrieved on (25 September 2024)	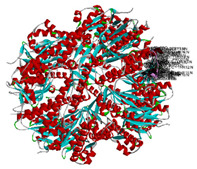 Link Retrieved on (25 September 2024)	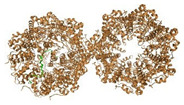 Link Retrieved on (26 September 2024)	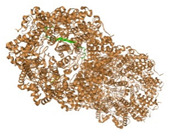 Link Retrieved on (26 September 2024)	−235.844 Single	−507.438 Single
MetG	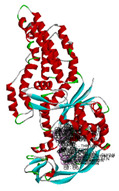 Link Retrieved on (25 September 2024)	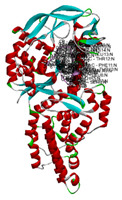 Link Retrieved on (25 September 2024)	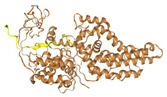 Link Retrieved on (26 September 2024)	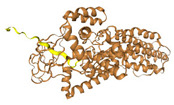 Link Retrieved on (26 September 2024)	−238.677 Single	−235.14 Single
FtsZ	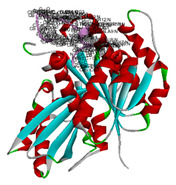 Link Retrieved on (25 September 2024)	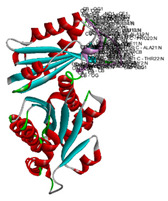 Link Retrieved on (25 September 2024)	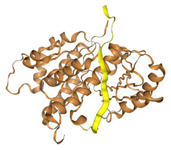 Link Retrieved on (26 September 2024)	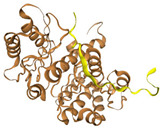 Link Retrieved on (26 September 2024)	−173.444 Single	−186.951 Single

**Table 3:** Predicted bacterial protein targets and putative mechanisms of action of AtMP2-derived peptides (based on docking simulations and literature reports) [25,26,27,28,29,30,31].

Gene/ Protein	Role in Bacterial Cell	Cell Cycle/ Process	Putative Mechanism (Based on Docking Predictions and Literature)	Reference
GyrA	Involved in DNA replication; mutations can lead to resistance	DNA Replication	Disrupts DNA replication, leading to bacterial cell death	[25]
RpoB	Part of RNA polymerase; mutations can affect transcription and antibiotic resistance	Transcription	Interferes with transcription, reducing bacterial protein production	[26]
SecA	Involved in protein translocation across membranes	Protein Transport	Disrupts protein transport, impairing cell functionality	[27]
GyrB	Assists in DNA replication; plays a role in cell division	DNA Replication and Cell Division	Disrupts DNA replication, affecting cell division and survival	[25]
GroEL	Chaperone protein involved in protein folding; essential for cell survival	Protein Folding	Inhibits protein folding, leading to the accumulation of misfolded proteins	[28]
ParE	Involved in DNA replication and repair; contributes to bacterial growth	DNA Replication and Repair	Interferes with DNA repair mechanisms, reducing bacterial viability	[26]
DnaK	Chaperone protein aids in stress response and protein refolding	Stress Response and Protein Refolding	Disrupts stress response, preventing refolding of damaged proteins	[28]
ClpP	Protease that degrades misfolded proteins, crucial for bacterial survival	Protein Quality Control	Inhibits protease activity, leading to the accumulation of damaged proteins	[29]
MetG	Involved in protein synthesis, specifically tRNA synthetase activity	Protein Synthesis	Impairs protein synthesis, reducing bacterial growth	[30]
FtsZ	Key player in bacterial cell division, forming the Z-ring at the site of division	Cell Division	Disrupts cell division by preventing the proper formation of the Z-ring	[31]

## Data Availability

Data is contained within the article or Supplementary Materials.

## Supplementary Materials

The following supporting information can be downloaded at: https://www.mdpi.com/article/10.3390/molecules30234590/s1, Table S1: Full protein names and their corresponding PDBID; Table S2: Docking scores and bond types of AtMP2-1 and AtMP2-2 peptides against proteins involved in bacterial cell death cycle.

## References

[1] Larsson D.G.J., Flach C.F. (2022). Antibiotic resistance in the environment. Nat. Rev. Microbiol..

[2] Lathakumari R.H., Vajravelu L.K., Satheesan A., Ravi S., Thulukanam J. (2024). Antibiotics and the gut microbiome: Understanding the impact on human health. Med. Microecol..

[3] Uddin T.M., Chakraborty A.J., Khusro A., Zidan B.R.M., Mitra S., Bin Emran T., Dhama K., Ripon K.H., Gajdács M., Sahibzada M.U.K. (2021). Antibiotic resistance in microbes: History, mechanisms, therapeutic strategies and prospects. J. Infect. Public Health.

[4] Huan Y., Kong Q., Mou H., Yi H. (2020). Antimicrobial Peptides: Classification, Design, Application and Research Progress in Multiple Fields. Front. Microbiol..

[5] Lin F.Y., Subramaniam G., Ambigai L., Ong G.H. (2021). Understanding the Use of Antibiotics and Antibiotic Resistance among Science Stream and Non-Science Stream Undergraduate Students in a Malaysian University. J. Liaquat Univ. Med. Health Sci..

[6] Moretta A., Scieuzo C., Petrone A.M., Salvia R., Manniello M.D., Franco A., Lucchetti D., Vassallo A., Vogel H., Sgambato A. (2021). Antimicrobial Peptides: A New Hope in Biomedical and Pharmaceutical Fields. Front. Cell. Infect. Microbiol..

[7] Nijnik A., Hancock R. (2009). Host defence peptides: Antimicrobial and immunomodulatory activity and potential applications for tackling antibiotic-resistant infections. Emerg. Health Threat. J..

[8] Mahlapuu M., Håkansson J., Ringstad L., Björn C. (2016). Antimicrobial Peptides: An Emerging Category of Therapeutic Agents. Front. Cell. Infect. Microbiol..

[9] Al-Rasheed A., Handool K.O., Alhelli A.M., Garba B., Muhialdin B.J., Masomian M., Hani H., Daud H.H.M. (2020). Assessment of Some Immune Components from The Bioactive Crude Extract Derived from The Epidermal Mucus of Climbing Perch *Anabas Testudines*. Turk. J. Fish Aquat. Sci..

[10] Najm A.A.K., Azfaralariff A., Dyari H.R.E., Othman B.A., Shahid M., Khalili N., Law D., Alwi S.S.S., Fazry S. (2021). Anti-breast cancer synthetic peptides derived from the *Anabas testudineus* skin mucus fractions. Sci. Rep..

[11] Reygaert W. (2018). An overview of the antimicrobial resistance mechanisms of bacteria. AIMS Microbiol..

[12] Mba I.E., Nweze E.I. (2022). Antimicrobial Peptides Therapy: An Emerging Alternative for Treating Drug-Resistant Bacteria. Yale J. Biol. Med..

[13] Chung C.R., Kuo T.R., Wu L.C., Lee T.Y., Horng J.T. (2020). Characterization and identification of antimicrobial peptides with different functional activities. Brief Bioinform..

[14] Hossain T.J. (2024). Methods for screening and evaluation of antimicrobial activity: A review of protocols, advantages, and limitations. Eur. J. Microbiol. Immunol..

[15] Oyama L.B., Olleik H., Teixeira A.C.N., Guidini M.M., Pickup J.A., Hui B.Y.P., Vidal N., Cookson A.R., Vallin H., Wilkinson T. (2022). In silico identification of two peptides with antibacterial activity against multidrug-resistant *Staphylococcus aureus*. NPJ Biofilms Microbiomes.

[16] Tincho M.B., Morris T., Meyer M., Pretorius A. (2020). Antibacterial Activity of Rationally Designed Antimicrobial Peptides. Int. J. Microbiol..

[17] Bahar A., Ren D. (2013). Antimicrobial Peptides. Pharmaceuticals.

[18] He S., Deber C.M. (2024). Interaction of designed cationic antimicrobial peptides with the outer membrane of Gram-negative bacteria. Sci. Rep..

[19] Malanovic N., Lohner K. (2016). Antimicrobial Peptides Targeting Gram-Positive Bacteria. Pharmaceuticals.

[20] Zhang Q.-Y., Yan Z.-B., Meng Y.-M., Hong X.-Y., Shao G., Ma J.-J., Cheng X.-R., Liu J., Kang J., Fu C.-Y. (2021). Antimicrobial peptides: Mechanism of action, activity and clinical potential. Mil. Med. Res..

[21] Theansungnoen T., Maijaroen S., Jangpromma N., Yaraksa N., Daduang S., Temsiripong T., Daduang J., Klaynongsruang S. (2016). Cationic Antimicrobial Peptides Derived from *Crocodylus siamensis* Leukocyte Extract, Revealing Anticancer Activity and Apoptotic Induction on Human Cervical Cancer Cells. Protein J..

[22] Kamiloglu S., Sari G., Ozdal T., Capanoglu E. (2020). Guidelines for cell viability assays. Food Front..

[23] Khalef L., Lydia R., Filicia K., Moussa B. (2024). Cell viability and cytotoxicity assays: Biochemical elements and cellular compartments. Cell Biochem. Funct..

[24] Macari G., Toti D., Pasquadibisceglie A., Polticelli F. (2020). DockingApp RF: A State-of-the-Art Novel Scoring Function for Molecular Docking in a User-Friendly Interface to AutoDock Vina. Int. J. Mol. Sci..

[25] Sada M., Kimura H., Nagasawa N., Akagawa M., Okayama K., Shirai T., Sunagawa S., Kimura R., Saraya T., Ishii H. (2022). Molecular Evolution of the *Pseudomonas aeruginosa* DNA Gyrase *gyrA* Gene. Microorganisms.

[26] Li M.-c., Lu J., Lu Y., Xiao T.-y., Liu H.-c., Lin S.-q., Xu D., Li G.-l., Zhao X.-q., Liu Z.-g. (2021). *rpoB* Mutations and Effects on Rifampin Resistance in *Mycobacterium tuberculosis*. Infect. Drug Resist..

[27] Gupta R., Toptygin D., Kaiser C.M. (2020). The SecA motor generates mechanical force during protein translocation. Nat. Commun..

[28] Fourie K.R., Wilson H.L. (2020). Understanding GroEL and DnaK Stress Response Proteins as Antigens for Bacterial Diseases. Vaccines.

[29] Nouri K., Feng Y., Schimmer A.D. (2020). Mitochondrial ClpP serine protease: Biological function and emerging target for cancer therapy. Cell Death Dis..

[30] Yi H., Lee H., Cho K.H., Kim H.S. (2018). Mutations in MetG (methionyl-tRNA synthetase) and TrmD [tRNA (guanine-N1)-methyltransferase] confer meropenem tolerance in *Burkholderia thailandensis*. J. Antimicrob. Chemother..

[31] Xiao J., Goley E.D. (2016). Redefining the roles of the FtsZ-ring in bacterial cytokinesis. Curr. Opin. Microbiol..

[32] Roca J. (2011). Transcriptional inhibition by DNA torsional stress. Transcription.

[33] Drlica K., Zhao X., Malik M. (2009). Quinolones. Encyclopedia of Microbiology.

[34] Dwyer D.J., Kohanski M.A., Hayete B., Collins J.J. (2007). Gyrase inhibitors induce an oxidative damage cellular death pathway in *Escherichia coli*. Mol. Syst. Biol..

[35] Ruan S., Tu C.H., Bourne C.R. (2024). Friend or Foe: Protein Inhibitors of DNA Gyrase. Biology.

[36] Vasilyev N., Liu M.M.J., Epshtein V., Shamovsky I., Nudler E. (2024). General transcription factor from *Escherichia coli* with a distinct mechanism of action. Nat. Struct. Mol. Biol..

[37] Jia X., He X., Huang C., Li J., Dong Z., Liu K. (2024). Protein translation: Biological processes and therapeutic strategies for human diseases. Signal Transduct. Target Ther..

[38] Almanza A., Carlesso A., Chintha C., Creedican S., Doultsinos D., Leuzzi B., Luís A., McCarthy N., Montibeller L., More S. (2019). Endoplasmic reticulum stress signalling—From basic mechanisms to clinical applications. FEBS J..

[39] Miller M.A., Zachary Z.J. (2017). Mechanisms and Morphology of Cellular Injury, Adaptation, and Death. Pathologic Basis of Veterinary Disease.

[40] Ajmal M.R. (2023). Protein Misfolding and Aggregation in Proteinopathies: Causes, Mechanisms, and Cellular Response. Diseases.

[41] UniProt SYM_ECOLI Entry P00959. https://www.uniprot.org/uniprotkb/P00959/entry.

[42] Wang G., Li X., Wang Z. (2016). APD3: The antimicrobial peptide database as a tool for research and education. Nucleic Acids Res..

[43] Waghu F.H., Barai R.S., Gurung P., Idicula-Thomas S. (2016). CAMP R3: A database on sequences, structures and signatures of antimicrobial peptides: Table 1. Nucleic Acids Res..

[44] Taoyuan (TW): Department of Computer Science and Information Engineering, National Central University [Internet] AMPfun. http://fdblab.csie.ncu.edu.tw/AMPfun/index.html.

[45] Hudzicki J. (2009). Kirby-Bauer disk diffusion susceptibility test protocol. Am. Soc. Microbiol..

[46] Koca Ö. (2024). Microbiological Characteristics of *Bacillus subtilis* Species and their Relationship with Hospital Infections. Bacterial, Viral, Fungal, and Parasitic Coinfections.

[47] Lee Z.Z., Abraham R., O’dEa M., Harb A., Hunt K., Lee T., Abraham S., Jordan D. (2021). Validation of Selective Agars for Detection and Quantification of *Escherichia coli* Strains Resistant to Critically Important Antimicrobials. Microbiol. Spectr..

[48] Kourmouli A., Valenti M., van Rijn E., Beaumont H.J.E., Kalantzi O.-I., Schmidt-Ott A., Biskos G. (2018). Can disc diffusion susceptibility tests assess the antimicrobial activity of engineered nanoparticles?. J. Nanopart. Res..

[49] Mercer D.K., Torres M.D.T., Duay S.S., Lovie E., Simpson L., von Köckritz-Blickwede M., de la Fuente-Nunez C., O’NEil D.A., Angeles-Boza A.M. (2020). Antimicrobial Susceptibility Testing of Antimicrobial Peptides to Better Predict Efficacy. Front. Cell. Infect. Microbiol..

[50] Kowalska-Krochmal B., Dudek-Wicher R. (2021). The Minimum Inhibitory Concentration of Antibiotics: Methods, Interpretation, Clinical Relevance. Pathogens.

[51] Werner A. How to Do Serial Dilutions (Including calculations) [Internet]. https://www.integra-biosciences.com/global/en/blog/article/how-do-serial-dilutions-including-calculations.

[52] Orellana E., Kasinski A. (2016). Sulforhodamine B (SRB) Assay in Cell Culture to Investigate Cell Proliferation. Bio Protoc..

[53] Alsedfy M.Y., Ebnalwaled A.A., Moustafa M., Said A.H. (2024). Investigating the binding affinity, molecular dynamics, and ADMET properties of curcumin-IONPs as a mucoadhesive bioavailable oral treatment for iron deficiency anemia. Sci. Rep..

