# Supplementary material for: Directed Evolution of AtMP2 Peptide: Unlocking Enhanced Antibacterial Potential from Anabas testudineus
Source: Molecules. 2025 Nov 28;30(23):4590. doi: 10.3390/molecules30234590 (PMC12693613; doi:10.3390/molecules30234590)
Supplement: Supplementary file 1 [file molecules-30-04590-s001.zip › molecules-3974264-supplementary.pdf]

Table S1: Full protein names and their corresponding PDBID.

Abbreviation	Full Protein Name	PDBID	Biological Function
GyrA	DNA gyrase subunit A	1AB4	DNA replication, negative supercoiling
GyrB	DNA gyrase subunit B	1EI1	ATP-dependent DNA supercoiling
RpoB	RNA polymerase β-subunit	4JKR	Transcription elongation
SecA	Preprotein translocase ATPase	2VDA	Protein translocation across membrane
GroEL	Chaperonin GroEL	1KP8	Protein folding
ParE	DNA topoisomerase IV subunit B	1S16	Chromosome segregation
DnaK	Chaperone protein DnaK	2KHO	Stress response, protein refolding
ClpP	ATP-dependent Clp protease proteolytic subunit	1YG6	Protein degradation
MetG	Methionyl-tRNA synthetase	1PFV	Protein synthesis
FtsZ	Cell division protein FtsZ	1FSZ	Cell division, Z-ring formation

Table S2: Docking scores and bond types of AtMP2-1 and AtMP2-2 peptides against proteins involved in bacterial cell death cycle

Protein	ZDOCK Server		HPEPDOCK Server		Docking Score & Bond Type	Docking Score & Bond Type
	AtMP2-1 (TGTATSGLATFTLHTGSLAPAT)	AtMP2-2 (TGWATSGLATFTLHTGSLAPAT)	AtMP2-1 (TGTATSGLATFTLHTGSLAPAT)	AtMP2-2 (TGWATSGLATFTLHTGSLAPAT)	AtMP2-1 Retrieved on (25/09/2024)	AtMP2-2 Retrieved on (25/09/2024)
Peptide	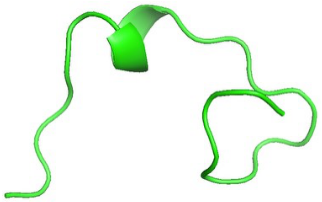	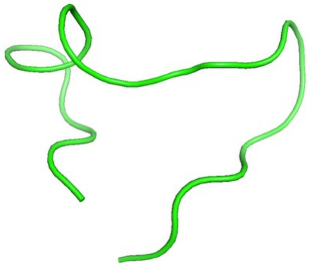				
GyrA	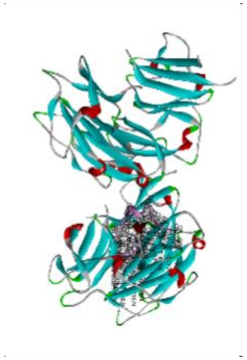 Link Retrieved on (25/09/2024)	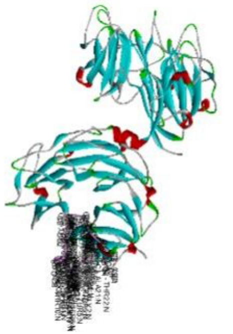 Link Retrieved on (25/09/2024)	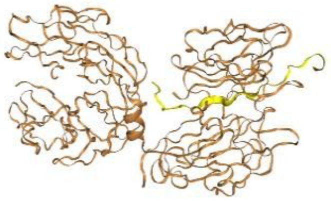 Link Retrieved on (25/09/2024)	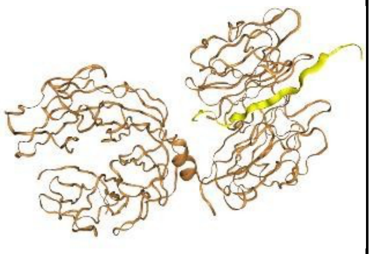 Link Retrieved on (26/09/2024)	-199.731 Single	-209.673 Single

GyrB	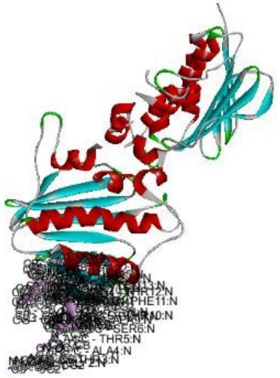 Link Retrieved on (25/09/2024)	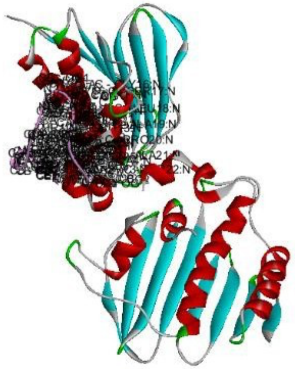 Link Retrieved on (25/09/2024)	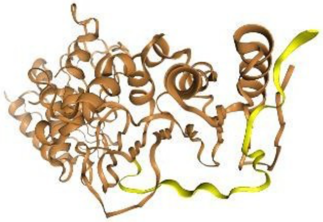 Link Retrieved on (25/09/2024)	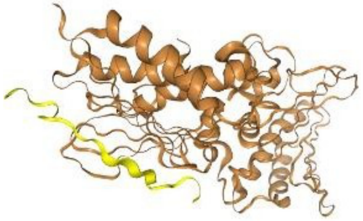 Link Retrieved on (26/09/2024)	-215.102 Single	-204.34 Single
RpoB	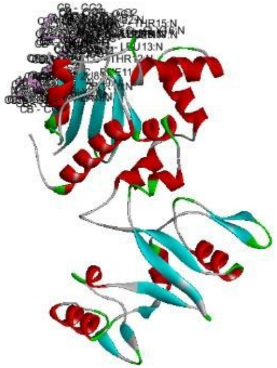 Link Retrieved on (25/09/2024)	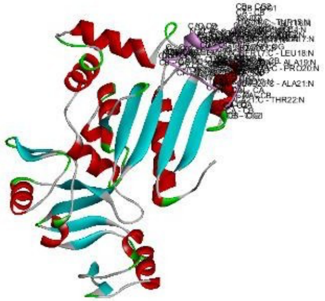 Link Retrieved on (25/09/2024)	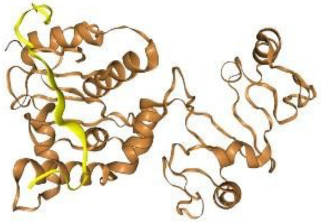 Link Retrieved on (25/09/2024)	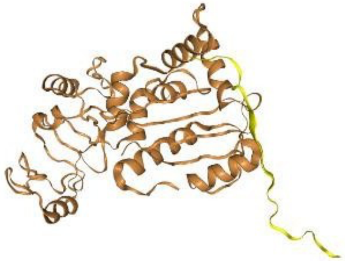 Link Retrieved on (26/09/2024)	-184.743 Single	-182.555 Single
SecA	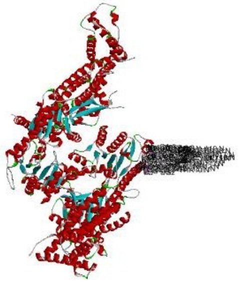 Link Retrieved on (25/09/2024)	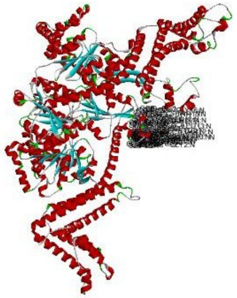 Link Retrieved on (25/09/2024)	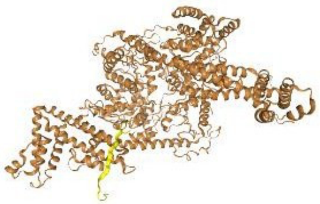 Link Retrieved on (25/09/2024)	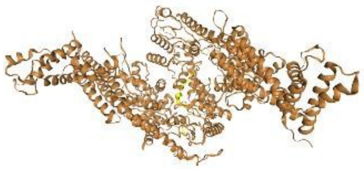 Link Retrieved on (26/09/2024)	-216.083 Single	-230.044 Single

GroEL	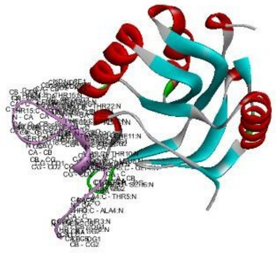 Link Retrieved on (25/09/2024)	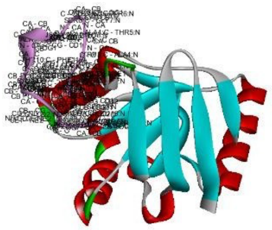 Link Retrieved on (25/09/2024)	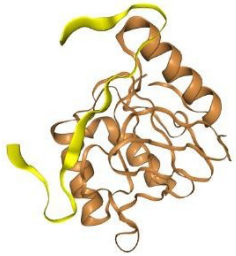 Link Retrieved on (25/09/2024)	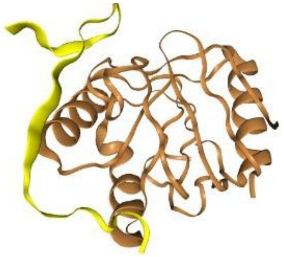 Link Retrieved on (26/09/2024)	-184.075<br>Single	-186.028<br>Single
ParE	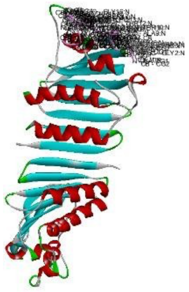 Link Retrieved on (25/09/2024)	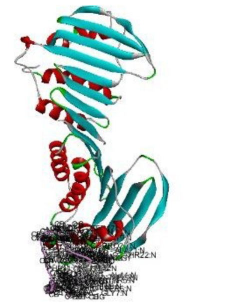 Link Retrieved on (25/09/2024)	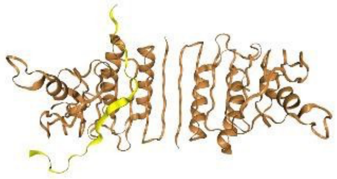 Link Retrieved on (25/09/2024)	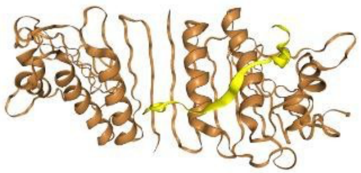 Link Retrieved on (26/09/2024)	-201.482<br>Single	-195.991<br>Single
DnaK	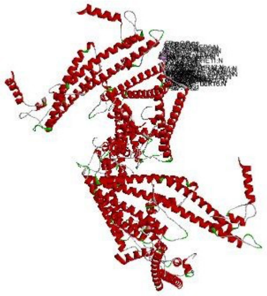 Link Retrieved on (25/09/2024)	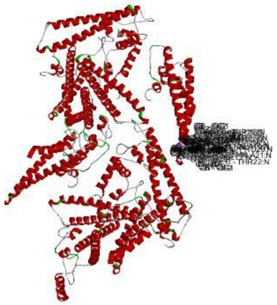 Link Retrieved on (25/09/2024)	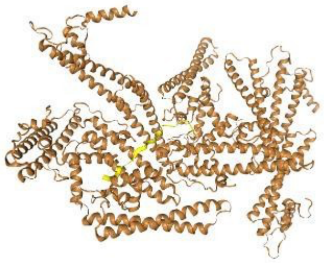 Link Retrieved on (26/09/2024)	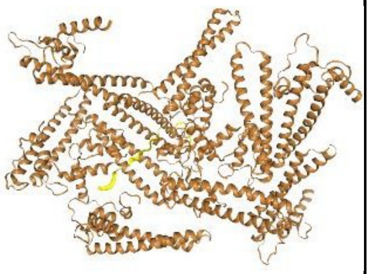 Link Retrieved on (26/09/2024)	-211.956<br>Single	-211.956<br>Single

ClpP	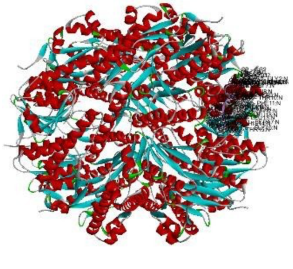 Link Retrieved on (25/09/2024)	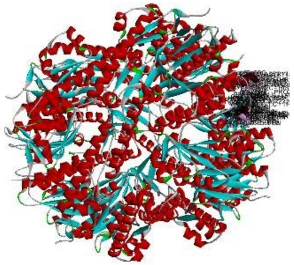 Link Retrieved on (25/09/2024)	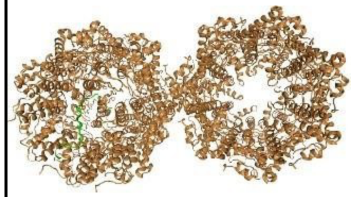 Link Retrieved on (26/09/2024)	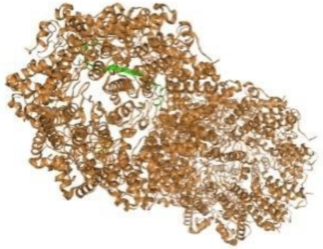 Link Retrieved on (26/09/2024)	-235.844 Single	-507.438 Single
MetG	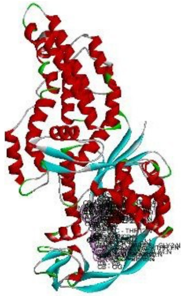 Link Retrieved on (25/09/2024)	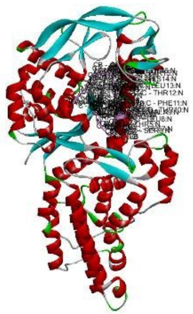 Link Retrieved on (25/09/2024)	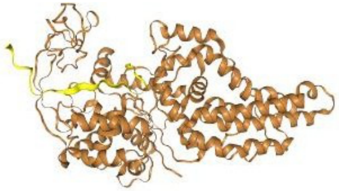 Link Retrieved on (26/09/2024)	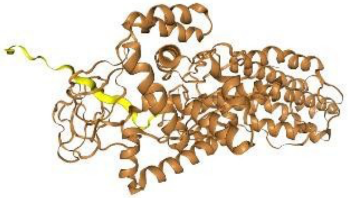 Link Retrieved on (26/09/2024)	-238.677 Single	-235.14 Single
FtsZ	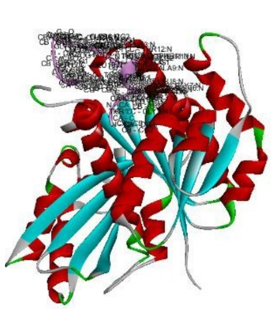 Link Retrieved on (25/09/2024)	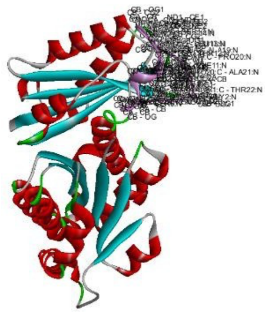 Link Retrieved on (25/09/2024)	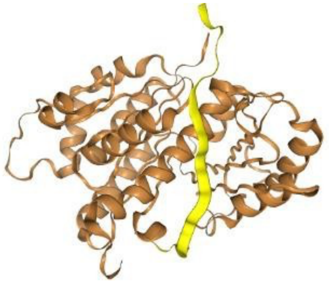 Link Retrieved on (26/09/2024)	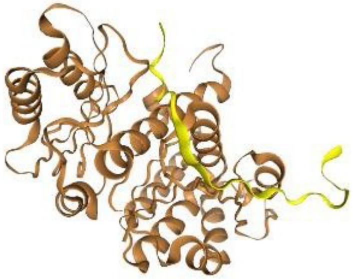 Link Retrieved on (26/09/2024)	-173.444 Single	-186.951 Single
